# Porcine deltacoronavirus nucleocapsid protein interacts with the Grb2 through its proline-rich motifs to induce activation of the Raf-MEK-ERK signal pathway and promote virus replication

**DOI:** 10.1099/jgv.0.002014

**Published:** 2024-08-13

**Authors:** Mingxia Li, Liping Zhang, Peng Zhou, Zhongwang Zhang, Ruiming Yu, Yongguang Zhang, Yonglu Wang, Huichen Guo, Li Pan, Sa Xiao, Xinsheng Liu

**Affiliations:** 1 State Key Laboratory for Animal Disease Control and Prevention, Key Laboratory of Animal Virology of Ministry of Agriculture, Lanzhou Veterinary Research Institute, Chinese Academy of Agricultural Sciences, Lanzhou, PR China; 2 College of Veterinary Medicine, Northwest A&F University, Yangling, Shaanxi, PR China

**Keywords:** Grb2, N protein, PDCoV, replication, ubiquitination

## Abstract

Porcine deltacoronavirus (PDCoV), an enteropathogenic coronavirus, causes severe watery diarrhoea, dehydration and high mortality in piglets, which has the potential for cross-species transmission in recent years. Growth factor receptor-bound protein 2 (Grb2) is a bridging protein that can couple cell surface receptors with intracellular signal transduction events. Here, we investigated the reciprocal regulation between Grb2 and PDCoV. It is found that Grb2 regulates PDCoV infection and promotes IFN-β production through activating Raf/MEK/ERK/STAT3 pathway signalling in PDCoV-infected swine testis cells to suppress viral replication. PDCoV N is capable of interacting with Grb2. The proline-rich motifs in the N- or C-terminal region of PDCoV N were critical for the interaction between PDCoV-N and Grb2. Except for *Deltacoronavirus* PDCoV N, the *Alphacoronavirus* PEDV N protein could interact with Grb2 and affect the regulation of PEDV replication, while the N protein of *Betacoronavirus* PHEV and *Gammacoronavirus* AIBV could not interact with Grb2. PDCoV N promotes Grb2 degradation by K48- and K63-linked ubiquitin-proteasome pathways. Overexpression of PDCoV N impaired the Grb2-mediated activated effect on the Raf/MEK/ERK/STAT3 signal pathway. Thus, our study reveals a novel mechanism of how host protein Grb2 protein regulates viral replication and how PDCoV N escaped natural immunity by interacting with Grb2.

## Introduction

Coronaviruses (CoVs) are a group of enveloped, single-stranded, positive-sense RNA viruses belonging to the family *Coronaviridae* that is within the order *Nidovirales*, which are genetically classified into four genera: *Alphacoronavirus*, *Betacoronavirus*, *Gammacoronavirus* and *Deltacoronavirus*. Porcine deltacoronavirus (PDCoV) is a novel swine enteropathogenic coronavirus belonging to *Deltacoronavirus* that causes diarrhoea and induces proinflammatory cytokine responses in piglets [1,3]. PDCoV has potential cross-species transmission in recent years [4]. PDCoV was first detected in pigs in Hong Kong in late 2012 [5], while the first outbreak of PDCoV-related diarrhoea in swine herds was reported in 2014 in the USA and rapidly spread in more than 20 states [6,8]. Subsequently, PDCoV was rapidly detected in many countries, including Canada [9], Mexico [10], South Korea [11], China [12], Japan [13], Lao PDR [14], Vietnam [15] and Thailand [16], posing a significant threat to the swine industry [17].

The PDCoV virion contains four major structural proteins, namely the N, M, E and S, and accessory proteins include NS6, NS7 and NS7a. Fifteen mature nonstructural proteins (nsps) are encoded by open reading frame 1a (ORF1a) and ORF1b. Although it is similar in general characteristics between PDCoV and those of other coronaviruses, the detailed functions of which are largely unknown in host cells [18]. The CoV N protein is highly conserved and the most abundant viral protein in infected cells, which is also involved in multiple stages of viral replication [19]. Additionally, the N protein plays a critical role in modulating inflammatory cytokines and counteracting the host’s innate immune [20,21]. Although N proteins from *Alphacoronavirus* and *Betacoronavirus* have been well studied, the functions of the *Deltacoronavirus* PDCoV in viral replication, pathogenesis and immune regulation remain unclear.

The growth factor receptor-bound protein 2 (Grb2), an adaptor molecule, is a key host factor in normal development and recruits some signalling molecules to participate in cellular responses, including cell growth, proliferation and metabolism [22,23]. The general functional domains of Grb2 consist of one central Src homology 2 (SH2) containing about 60–158 aa, and two flanking include the N-terminal and C-terminal Src homology 3 (SH3), respectively, containing approximately 5–55 aa and 164–214 aa [24,25]. The SH2 domain receives signals from upstream target proteins, and then the SH3 domain receives the signal from SH2 and transmits downstream, such as Grb2 provides critical links by carrying messages from receptor tyrosine kinases (RTKs) to downstream Ras protein [26]. Although previous studies have shown Grb2 proteins can interact with viral proteins to regulate viral replication [27,29], it is unclear whether Grb2 participates in the replication of coronaviruses.

The activity of Grb2 is signal transduction by activating RTKs and Ras-specific guanine nucleotide exchange factor (GEF) [30,31]. Grb2 acts as an adaptor to link growth factor receptors to the Mitogen-Activated Protein Kinase Kinase Kinase cascade in the extracellular signal-regulated kinases 1 and 2 (ERK1/2) pathway via Ras. Raf/MEK/ERK signal transduction pathway is one of the mitogen-active protein kinase (MAPK) cascades and comprises an array of three consecutive-acting kinases: Raf, MEK1/2 and ERK1/2. The MAPK pathways are central signalling networks that control some principal pathways [32]. Many viruses hijack and inherit the cellular signalling cascades to regulate their replication cycle [32,34].

The present study identifies the novel interaction between PDCoV N and Grb2 protein, which differs from other genera of coronavirus. In addition, PDCoV infection induces upregulated Grb2 expression and the activation of the Raf/MEK/ERK signal pathway. Overexpression of PDCoV N impaired the Grb2-mediated activated effect on the Raf/MEK/ERK signal pathway activation. Our findings provide molecular insight into the potential role of Grb2 in regulating PDCoV replication and showed a function of POCoV N during the interaction between *Deltacoronavirus* and the host.

## Methods

### Cells, viruses, plasmids and reagents

Porcine kidney (LLC-PK1) cells (ATCC, CL-101), swine testis (ST) cells (ATCC, CRL-1746) and African green monkey kidney (Vero) cells (ATCC, CCL-81) were obtained from the ATCC. LLC-PK1 and ST cells were cultured in Eagle Minimal Essential Medium, and Vero cells were in Dulbecco’s Modified Eagle Medium. They were all supplemented with 10 % fetal bovine serum at 37℃ under 5 % CO_2_. The knockdown cells named mST-1B9 were generated by Haixing company and grown in MEM. CH/XJYN/2016 (GenBank no: MN064712) and CH/HBXT/2018 (GenBank no: MH816969) were isolated and maintained in our laboratory. The genes for *Grb2* containing a Flag tag and *PDCoV-N* were constructed using a pcDNA3.1(+) vector in-frame and named pcDNA3.1-pGrb2, pcDNA3.1-mGrb2 and pcDNA3.1-PDN. The genes for SYVN1 were constructed using a pcDNA3.1-myc-his vector in-frame and named pcDNA3.1-SYVN1-myc-his. The *Grb2* gene was cloned into the pGEX-4T-1 vector to construct plasmid encoding GST-tagged. We also cloned the sequences of PDCoV-N named wPDCoV-N to pet24a vector to express and purify His-fused protein. The mutant plasmids mPDCoV-N (P61A), mPDCoV-N (P292A) and mPDCoV-N (P61A/P292A) were generated using a kit according to the instructions of the manufacturer (QuikChange Lightning Site-Directed Mutagenesis Kit). The integrity of all constructs was confirmed by sequencing. Ubiquitin-related plasmids such as pRK5-HA-Ubiquitin-WT and pRK5-HA-Ubiquitin-K6/K11/K27/K29/K33/K48/K63 were purchased from Miaoling Biology. The PDCoV and PEDV stocks were propagated in LLC-PK1 and Vero cells. The viral litres were determined as the 50% tissue culture infectious dose.

### Transfections and real-time PCR assays

The cells in 35 cm^2^ dishes were transfected with various plasmids using Lipofectamine 3000 according to the manufacturer’s protocol and infection with CH/XJYN/2016 at a multiple of infection (MOI) of 0.01 for 18 h. mRNA expression levels of *Grb2* and *PDCoV-N* were quantitated by real-time quantitative Polymerase Chain Reaction (RT-qPCR). Total RNA was extracted from cells using TRizol according to the manufacturer’s protocol. RT-qPCR was performed with SYBR green or Taq real-time PCR master mixes. β-Actin was used as the reference gene, and SYBR green real-time PCR master mix data were determined by the threshold cycle (2^−ΔCT^) method. Taq real-time PCR was measured by a standard curve established in our laboratory, with the results expressed as relative fold change. Primers for RT-PCR are available upon request.

### Western blotting

The cell was lysed with Radio-Immunoprecipitation Assay Lysis Buffer. Clarified cell lysates were diluted in denaturing SDS gel loading buffer and boiled for 10 min. After denaturing, the samples were separated on a 10 % SDS-PAGE gel and blotted onto the PVDF membrane. The membrane was blocked with 5 % skim milk in Tris-Buffered Saline Tween, followed by incubation with the following primary antibodies: rabbit anti-Grb2 mAb (1 : 2000; Abcam), mouse anti-Flag, anti-HA and anti-myc pAb (1 : 2000, Abmart), mouse anti-PDN and anti-PEN MAb (1 : 5000, own laboratory), rabbit anti-PDM pAb (1 : 500, own laboratory), mouse anti-GST pAb (1 : 2000, Abmart), mouse anti-β-tubulin and anti-β-actin mAb (1 : 4000, Zhongshan Golden Bridge Biotechnology), rabbit anti-ERK1/2 and anti-pERK1/2 mAb (1 : 1000, Cell Signalling Technology) and rabbit anti-STAT3 and anti-pSTAT3 (1 : 2000, Abmart). After washing, the membrane was incubated with horseradish peroxidase-conjugated goat anti-rabbit pAb antibody or goat anti-mouse pAb antibody (1 : 5000, Zhongshan Golden Bridge Biotechnology) and detected by using enhanced chemiluminescence reagent.

### Coimmunoprecipitation assay

Cells were transfected using Lipo 3000 (Invitrogen) according to the manufacturer’s instructions. pcDNA3.1-pGrb2 was transfected into LLC-PK1 cells. After 24 h post-transfection, cells were infected PDCoV at 15 h post-infection (hpi) at 0.01 MOI. The cell was lysed with NP40 for 1 h at 4 ℃. The supernatant was collected and incubated overnight with anti-Flag or anti-PDN antibodies. After that, the supernatant was incubated with protein A+G agarose beading (Beyotime) for 6 h at 4 ℃. After six washes with prechilled PBS buffer, the beads were resuspended and subjected to SDS-PAGE.

For mass spectrometry identification of pcDNA3.1-pGrb2 or pcDNA3.1-PDN interacting proteins, one 100 cm^2^ dish of LLC-PK1 cells was useful to transfected. The protein samples were extracted as described above and were analysed using mass spectrometry by Lanzhou University.

### Protein purification

Glutathione S-transferase (GST) and GST-fused proteins were expressed in BL21(DE3) *Escherichia coli* cells and purified according to the manufacturer’s protocols (GenScript). His-tagged proteins wPDCoV-N, mPDCoV-N(P61A), mPDCoV-N(P292A) and mPDCoV-N(P61/292A) were expressed in BL21(DE3). Crude lysate was incubated with Ni +according to the manufacturer’s instructions (GenScript). After cells were washed with the washing buffer (20 mM Tris-HCL, 200 mM NaCl, 50 mM imidazole, pH 8.0), proteins were eluted with 200 mM imidazole.

### GST-pull down assay

The purified GST-tagged, including pGEX-Grb2 (Grb2-GST) and pGEX-4T-1 (GST), were mixed with prokaryotic expression and purified His-fused coronavirus N proteins overnight at 4 ℃. All supernatants were transferred to GST medium (Invitrogen) for 4 h at 4 ℃. After a washing step, the prechilled elution buffer was used to elute GST protein. The supernatants were responded to and subjected to SDS-PAGE.

### Confocal microscopy

ST cells were seeded on a glass bottom cell dish for 12 h before transfection. Cells were transfected with pcDNA3.1-pGrb2. After 24 h post-transfection, the cells were infected with CH/XJYN/2016 for 15 h. After that, the cells were fixed with 4 % paraformaldehyde. The cells were then permeabilized with 0.1 % Triton X-100 and blocked with 1 % BSA. The cells were incubated with rabbit anti-Grb2 mAb (1 : 2000) and mouse anti-PDN MAb (1 : 2000) primary antibody for 2 h at room temperature. After three washes with PBS, FITC-conjugated goat anti-mouse or TRITC-conjugated goat anti-rabbit secondary (1 : 2000) were added, followed by incubation for 1 h at room temperature. After another three washes with PBS, the cells were stained with DAPI (1 : 2000) for 10 min and washed three times with PBS at room temperature before being observed under a confocal microscope.

### Quartz crystal microbalance test (QCM)

The fresh-coated quartz crystal was mounted in the flow-through system and continuously rinsed with PBS (pH 7.4) until the frequency stabilized under flow conditions (60 μl/min). Various protein solutions (purified N proteins) were injected into the fluid system by an injection valve. The permanent frequency shifts versus time curves were recorded, and the binding process was monitored in real time. The first binding measurement was finished until the frequency had stabilized under flow conditions. After one binding measurement, Tris-HCl solution (pH 8.0) was injected into the system with the injection valve to dissociate the free proteins for the next binding. The second binding measurement was GST protein was injected into the fluid system. The frequency shifts did not change as protein solutions did. Tris-HCl solution (pH 8.0) was injected into the system and used to dissociate the free proteins. The third binding measurement was the Grb2-GST protein. The step was the same as above. The permanent frequency shifts versus time curves were recorded, and the binding process was monitored in real time. The binding ratio was calculated between the amplitude difference of Grb2-GST protein before and after binding and the amplitude of N protein in the early stage.

## Results

### PDCoV infection upregulates Grb2 expression

**Fig. 1.**
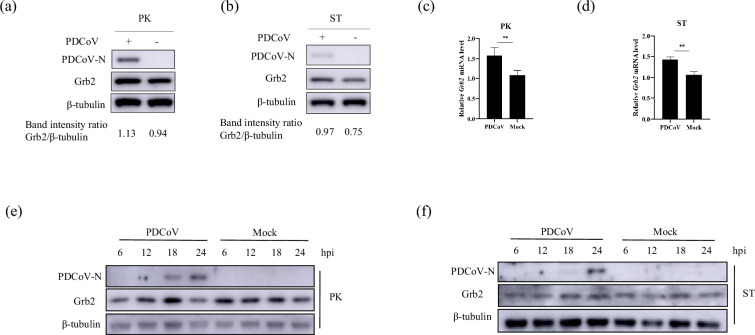
PDCoV CH/XJYN/2016 virus infection induced the upregulation of the Grb2 protein. (**a–d**) Western blotting and RT-qPCR analysis of Grb2 and PDCoV N in LLC-PK1 and ST cells by PDCoV infection (MOI = 0.01) at 18 hpi. The level of Grb2 in whole-cell lysates was quantitated by band intensities analysed using Image J (NIH). LLC-PK1 (**e**) and ST (**f**) cells were infected PDCoV with MOI = 0.01. Cell lysates were collected and assayed for expression. All experiment results are presented as the mean±SD from three independent experiments (*P≤0.05; **P≤0.01; all by two-tailed Student’s *t*-test).

The differential expression of proteins in PDCoV-infected and mock-infected LLC-PK1 cells at 18 hpi was analysed by liquid chromatography-tandem mass spectrometry combined with tandem mass tag (TMT) labelling in our previous study. The results showed that Grb2 was upregulated after PDCoV infection [35]. To verify the previous results, the expression level of Grb2 in PDCoV-infected LLC-PK1 and ST cells with the MOI of 0.01 were assessed. The results showed that Grb2 was significantly upregulated on mRNA and protein levels in LLC-PK1 (Fig. 1a, c) and ST (Fig. 1b, d) cells during PDCoV infection at 18 hpi. Further, LLC-PK1 and ST cells were infected with PDCoV (MOI = 0.01) and harvested at the indicated times. The cell lysates were subjected to Western blotting. The expression level of Grb2 was significantly increased from 6 to 18 hpi but reduced at 24 hpi in LLC-PK1 (Fig. 1e). Besides that, the expression level of Grb2 was significantly increased from 6 to 24 hpi in ST (Fig. 1f), indicating that expression of Grb2 was upregulated at the early stage during PDCoV infection. Overall, the Grb2 expression level could be regulated by PDCoV infection.

### Overexpression of Grb2 inhibits PDCoV replication with a dose-dependent manner

**Fig. 2.**
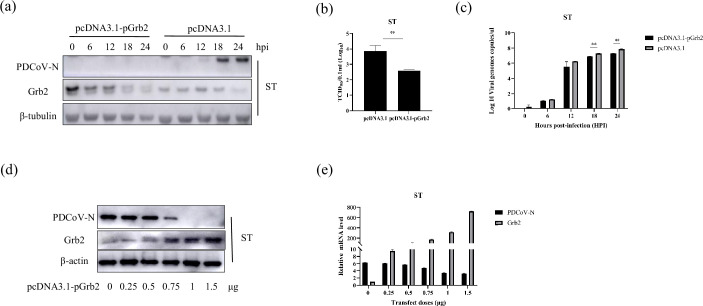
Grb2 inhibited PDCoV replication in ST cells. (**a–c**) ST cells were transfected with a plasmid encoding Flag-pGrb2, or the empty vector for 24 h, and then infected with the PDCoV at an MOI of 0.01, followed by Western blotting and RT-qPCR. The cell culture supernatants were collected and assayed for virus litres by TCID_50_. (**d and e**) ST cells were transfected with increasing concentrations of a vector expressing pcDNA3.1-pGrb2. At 24 h post-transfection, the cells were infected with PDCoV at an MOI of 0.01 and then analysed PDCoV N and Grb2 expression level.

To investigate whether Grb2 participates in the replicate regulation of PDCoV, the ST cells were transfected with a plasmid encoding flag tag and full-length Grb2 (pcDNA3.1-pGrb2) or empty vector plasmid for 24 h and then infected with PDCoV with 0.01 MOI. The resultant lysates were assessed by Western blotting (Fig. 2a), RT-qPCR (Fig. 2c) and the median tissue culture infective dose (TCID_50_) (Fig. 2b). The results showed that the PDCoV N protein and mRNA levels were both significantly decreased by Grb2-overexpressing ST cells compared with their levels in empty vector plasmid transfected cells. Besides, the TCID_50_ was decreased from 3.85 to 2.59. The results revealed that Grb2 expression effectively abolished PDCoV replication. Further, ST cells were transfected with increasing concentrations of pcDNA3.1-pGrb2, and were infected with PDCoV at an MOI of 0.01 at 18 h. It was found that Grb2 significantly inhibited virus replication in a dose-dependent manner (Fig. 2d, e). Together, these results indicated that the overexpression of Grb2 inhibited PDCoV replication in ST cells.

### Knockdown of Grb2 increases PDCoV replication in ST

**Fig. 3.**
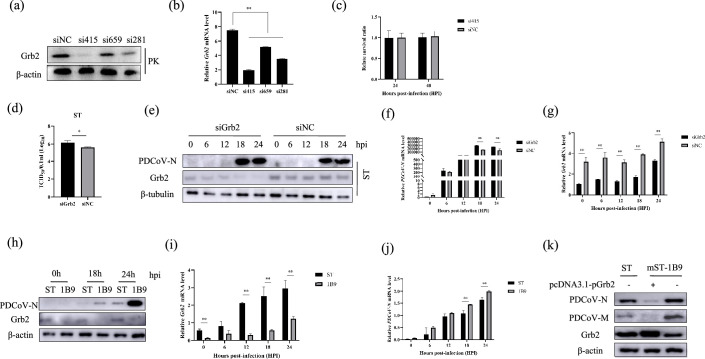
(**a and b**) LLC-PK1 cells were transfected with three Grb2-specific siRNAs. Grb2 expression was assessed. (**c**) LLC-PK1 cells were transfected with si415 or siNC for 24 h and 48 h. The cell viability was measured by MTT assay. (**d–g**) ST cells were transfected with siGrb2 or siNC. The cells were infected with PDCoV at 24 h followed by Western blotting, RT-qPCR and TCID_50_. (**h–j**) The WT ST cell and its mutants with knockdown of Grb2 named mST-1B9 were infected PDCoV. The expression of PDCoV-N was analysed. (**k**) The ST was transfected the pcDNA3.1, and mST-1B9 cells were transfected pcDNA3.1-pGrb2 or pcDNA3.1, followed by infection with the PDCoV. The cell lysates were collected to analyse PDCoV N, M and Grb2 protein expression.

To further evaluate the effect of Grb2 on PDCoV replication, three short interfering RNA (siRNA) targeting pGrb2 were designed and synthesized. The knockdown efficiency of three siRNA was tested by Western blotting and RT-qPCR (Fig. 3a, b). The results showed that Grb2 expression was significantly lower in cells transfected with siRNA-415 than in cells transfected with non-targeting siRNA (siNC) at 24 h. siRNA-415 had no effect on cellular activity, which could be used for follow-up experiments (Fig. 3c). The ST cells were transfected siGrb2 for 24 h and infected with PDCoV. The viral replication was analysed by Western blotting (Fig. 3e), RT-qPCR (Fig. 3f, g) and TCID_50_ (Fig. 3d). Downexpression of Grb2 significantly increased PDCoV N protein abundance in ST cells during PDCoV infection. Viral RNA detection also revealed that decreased expression of Grb2 promoted PDCoV replication. The TCID_50_ showed the upregulation was statistically significant. The above results showed that the knockdown of Grb2 markedly increased the virus replication at 18 and 24 hpi.

Furthermore, the Grb2 knockdown ST (mST-1B9) cell lines were constructed. The WT cells and mST-1B9 were infected PDCoV and harvested at indicate time points. The RT-qPCR (Fig. 3i, j) and Western blotting (Fig. 3h) results showed that the knockdown of Grb2 increased the virus replication in the ST cell. The pcDNA3.1-pGrb2 was transfected into mST-1B9 cells and then infected with PDCoV. On the basis of the rise of plasmid (pcDNA3.1-pGrb2) quantity, the PDCoV N and M protein levels notably lowered. The results showed that PDCoV replication was inhibited after overexpression of Grb2 in mST-1B9 cells (Fig. 3k). Downregulation of Grb2 significantly promoted PDCoV replication in ST.

### Grb2 promote production of IFN-β through activates Raf/MEK/ERK/STAT3 pathway signalling in PDCoV-infected cells to suppress viral replication

**Fig. 4.**
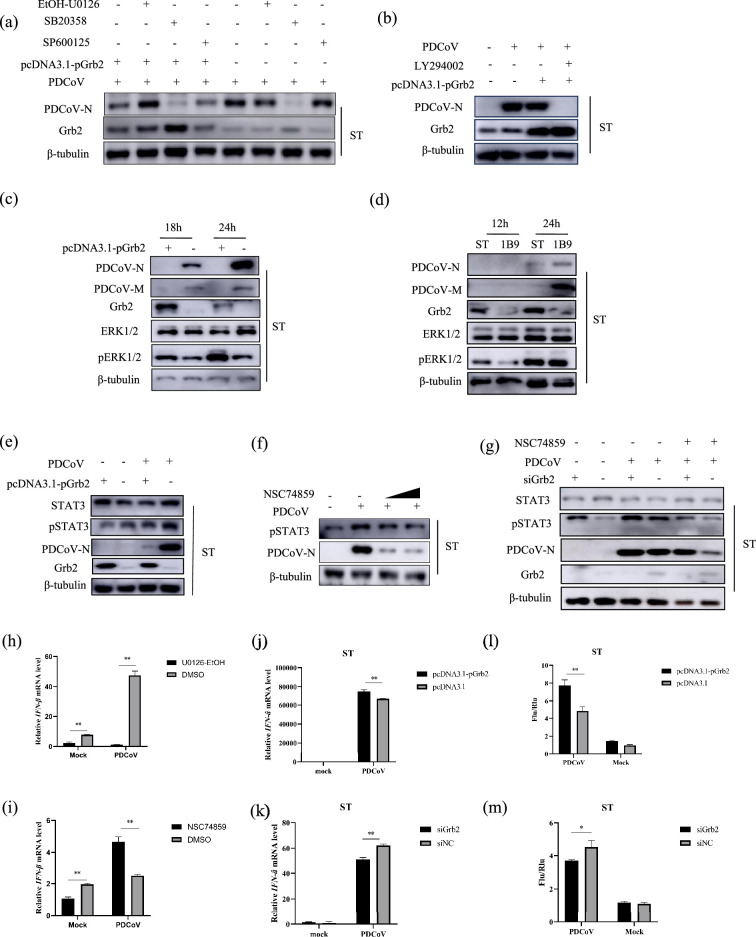
Grb2 increased PDCoV-induced IFN-β activation. (**a and b**) ST cells were transfected with pcDNA3.1-pGrb2 or pcDNA3.1 for 24 h and then were pretreated with DMSO (solvent control), SB203580 (5 μM), SP600125 (1 μM), U0126-EtOH (10 µM) or LY294002 (10 µM) for 2 h. After that, cells were infected with PDCoV for 18 h in the presence of each inhibitor of the MAPK pathway. PDCoV N production was measured. (**c**) ST cells that overexpress Grb2 were infected with PDCoV at 24 h. (**d**) ST and mST-1B9 were infected PDCoV; the cell lysates were collected for analysis expression of ERK1/2 and phosphorylated ERK1/2 (pERK1/2). (**e**) ST cells were transfected with pcDNA3.1-pGrb2 and infected with PDCoV; STAT3 activity was analysed. (**f**) ST cells were treated with STAT3 inhibitor (NSC74859) with different concentrations and were measured by Western blotting. (**g**) ST cells were transfected with siGrb2 or siNC and then pretreated with DMSO or NSC74859 for 2 h. After that, cell was infected with PDCoV for 18 h in the presence of an inhibitor. PDCoV N production was measured. The inhibitors U0126-EtOH (**h**) and NSC74859 (**i**) were used to treat ST cells. The cells were infected with PDCoV or not. The mRNA levels of IFN-β were assayed. ST cells were overexpressed with pcDNA3.1-pGrb2 (**j**) or siGrb2 (**k**). The cells were infected with PDCoV. IFN-β mRNA levels were analysed at 12 hpi. ST cells were cotransfected with IFN-β-Luc, pRL-TK, and the other indicated expression plasmids, including pcDNA3.1-pGrb2 (**l**) or siGrb2 (**m**). Cells were stimulated with PDCoV or were left untreated for 12 h. A dual-Luciferase assay was then performed.

As mentioned, Grb2 is a key adaptor protein upstream of the Raf/MEK/ERK and PI3K/Akt pathway. To investigate this possibility and to determine which key pathway the Grb2 displays its activity. Four inhibitors named U0126-EtOH, SB203580, SP600125 (Fig. 4a) and LY294002 (Fig. 4b) were used to treat Grb2-overexpressed cells. The expression level of PDCoV N protein was observably increased in the group receiving U0126-EtOH treatment accompanied by a decline of pERK1/2, indicating that the activation of Raf/MEK/ERK pathway signalling could inhibit PDCoV replication. The above results indicated that Grb2 suppresses PDCoV replication by activating the Raf/MEK/ERK pathway.

Furthermore, to evaluate whether Grb2 regulated Raf/MEK/ERK pathway (Fig. 4c), pcDNA3.1-pGrb2 were exposed to transfection into ST cells. After 24 h post-transfection, the cells were subject to infection with PDCoV at the MOI of 0.01. The level of ERK1/2, the phosphorylation of ERK1/2 (pERK1/2) and the N protein of PDCoV were measured at the indicated time points by Western blotting. We found that pERK1/2 was dramatically increased in Grb2-overexpressed ST cells, while the N protein of PDCoV was decreased. On the other hand, pERK1/2 was significantly reduced in Grb2 knockdown cells (Fig. 4d), while the N protein of PDCoV obviously increased. It indicats that Raf/MEK/ERK pathway signalling could be activated by Grb2 and affect virus replication.

According to previous studies, it has been proven that ERK1/2 inhibited the activation of STAT3. It was found that Grb2 significantly inhibited the phosphorylation of STAT3 in ST cells (Fig. 4e). Besides that, ST cells were treated with STAT3 specific inhibitor named NSC74859 showed dose-dependent inhibition of PDCoV replication, indicating that STAT3 positively regulated PDCoV replication (Fig. 4f). So, the inhibitor NSC74859 was used to treat siGrb2 overexpressed cells. As shown in Fig. 4g, the level of pSTAT3 protein was observably decreased in the group receiving NSC74859 treatment, accompanied by the decline of PDCoV N, indicating that the inactivation of pSTAT3 could inhibit PDCoV replication.

As is well known, STAT3 is a negative regulatory factor of IFN-β production. Subsequently, the inhibitors U0126-EtOH and NSC74859 were used to treat ST cells. The mRNA levels of IFN-β were measured. The results showed the level of IFN-β was downregulated and upregulated in cell treatment with U0126-EtOH (Fig. 4h) and NSC74859 (Fig. 4i), respectively. The pcDNA3.1-pGrb2 (Fig. 4j) or siGrb2 (Fig. 4k) were overexpressed in ST cells and after that infected with PDCoV. The mRNA levels of IFN-β were examined at 12 hpi. It was verified that the mRNA levels of IFN-β were upregulated and downregulated in Grb2 overexpression (Fig. 4l) cells and Grb2 knockdown (Fig. 4m) cells, respectively. Those results indicated that Grb2 could regulate IFN-β produce. The conclusion was further confirmed by the dual luciferase report system.

To further reveal the mechanism of Grb2-mediated anti-PDCoV effect, the cells were infected with PDCoV at the MOI of 0.01 after 24 h post-transfection of pcDNA3.1-pGrb2. Then, the cell lysates were harvested at 15 h, and a coimmunoprecipitation (CO-IP) assay with LC-MC/MS was performed using flag as the bait. As shown in Table S1 (available in the online Supplementary Material), most of the Grb2-trapped proteins were related to IFN-β induction, such as classical IFN stimulated genes (ISGs), OAS1, OASL, IFIT2 and Mx1. In light of this, it was speculated that Grb2 is involved in IFN-β production.

It is well-known that the transcription factors are mainly regulated by the Raf/MEK/ERK pathway. So, we speculated that Grb2 promotes the production of IFN-β by regulating the expression of host transcription factors. To further confirm the hypothesis, the proteins from CO-IP with LC-MC/MS were used to predict the transcription factor by TRRUST (Table 1). CREB5 and STAT3 were obtained. The STAT3 could regulate the expression of HSPB1, LGALS3BP and OAS1. This result further proved that STAT3 plays an important role in Grb2 regulation of PDCoV replication. Together, these results indicate that Grb2 promotes the production of IFN-β through activates Raf/MEK/ERK/STAT3 pathway signalling in PDCoV-infected ST cells to suppress viral replication.

**Table 1.** The transcription factors were predicated by TRRUST

Key TF	Description	Targets	*P* value	FDR
CREB5	Cam responsive element binding protein 5	LGALS3BP MX1	0.00016	0.000319
STAT3	Signal transducer and activator of transcription 3	HSPB1 LGALS3BP OAS1	0.00146	0.00146

P values are calculated with hypergeometric test.

The FDR was calculated using the Benjamini and Hochberg approach to adjust for multiple testing correction.

*http://www.grnpedia.org/trrust/

FDR, False discovery rate; TF, Transcription factor.

### The PDCoV-N protein interacts with Grb2

**Fig. 5.**
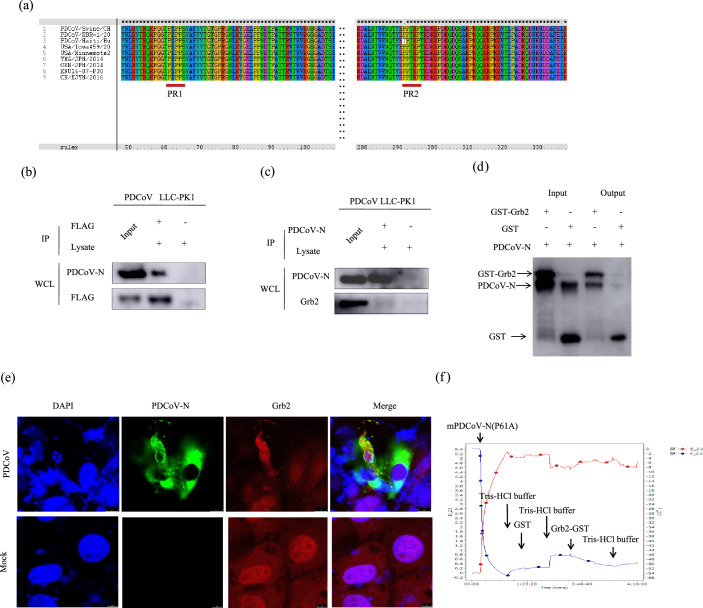
PDCoV N interacts with Grb2 protein directly. (**a**) PDCoV N protein sequences were from different strains including two American isolate strains USA/Iowa459/2014 and USA/Minnesota214/2014, two Japanese isolate strains YMG/JPN/2014 and OKN/JPN/2014, two Chinese isolate strains PDCoV/Swine/CHJX/2015 and CH/XJYN/2016 was from our laboratory, one Thailand isolate strain PDCoV/RBR-1/2016/Thailand, one Korera isolate strain KNU16-07-P30 and PDCoV/Haiti/Human was isolated from human. All sequences were comparative analysed by Clustal X. LLC-PK1 with overexpressed Grb2 were infected PDCoV CH/XJYN/2016 for 15 h. Co-IP with anti-Flag (**b**) and anti-PDCoV N (**c**) binding beads were finished. (**d**) The PDCoV N with 6×Hig-tagged and Grb2 with GST proteins were expressed and purified for the GST pull-down analysis. (**e**) The overexpressed Grb2 cells were infected PDCoV or not. The subcellular localization of Grb2 and PDCoV N was observed by confocal immunofluorescence microscopy. Anti-PDCoV N (green) and anti-Grb2 (red) antibodies and DAPI (blue) were used to stain. Scale bars: 10 µm. (**f**) The binding affinity between PDCoV-N and Grb2 was analysed by QCM.

It is known that Grb2 is an adaptor protein consisting of an SH2 domain flanked by N- and C-terminal SH3 domains and binds other proteins mainly through its SH3 domains to proline-rich (PR) motifs [30]. Hence, to investigate the PDCoV proteins that may interact with the Grb2, the full genome of PDCoV from different isolated strains was analysed, and the results showed that only N protein contains two conserved proline-rich motifs at 61 to 64 aa and 291 to 295 aa, respectively (Fig. 5a). According to previous results from CO-IP assay with LC-MC/MS (Table S1). PDCoV N was also using flag as the bait. It is suggested that PDCoV N may interact with Grb2. To verify the interaction of Grb2 with PDCoV N protein, the plasmids encoding Flag-Grb2 was transfected into LLC-PK1 cells for 24 h followed by PDCoV infection. Co-IP assay was performed using anti-Flag (Fig. 5b), anti-PDCoV N (Fig. 5c) or anti-IgG agarose beads. PDCoV N was coimmunoprecipitation with Flag-Grb2 and vice versa, indicating that there was an interaction between Grb2 and PDCoV N. Furthermore, GST pull-down assay was performed and confirmed the direct interaction between Grb2 and PDCoV-N (Fig. 5d). ST cells were transfected pcDNA3.1-pGrb2 and then were infected with PDCoV for 15 h. The confocal laser microscopy was employed to explore the colocalization of PDCoV-N and Grb2 during PDCoV infection (Fig. 5e). Besides, QCM was performed, which is a continuous, real-time detector for assessing the kinetics of interaction exists [36]. Its results showed that the amplitude curve had sharp fluctuations after adding Grb2-GST, but there was no change after adding GST (Fig. 5f). Collectively, it was shown that PDCoV N is capable of interacting with Grb2.

### Identification of the Grb2 interaction site on PDCoV N

**Fig. 6.**
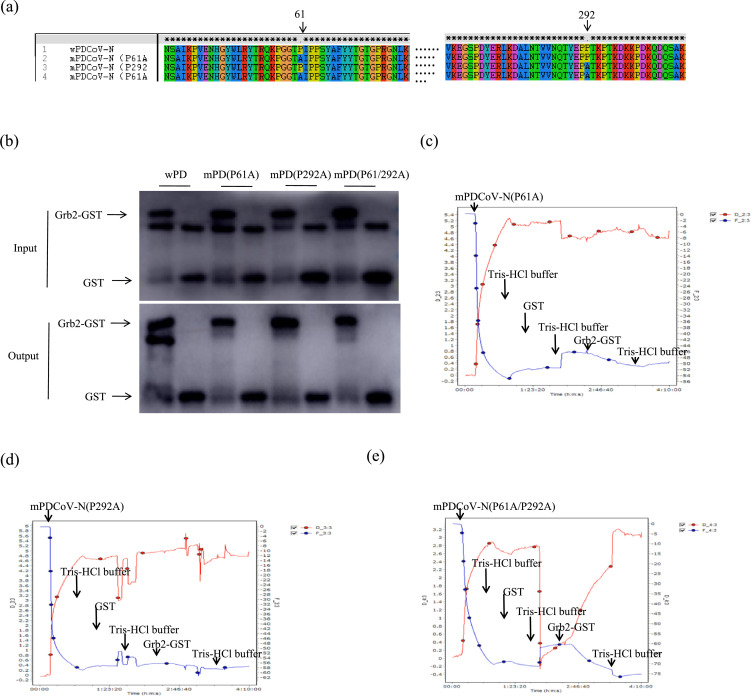
The 61–64 aa and 291–295 aa sites of PDCoV N protein interact with Grb2. (**a**) Strategy to construct the mutants. Mutations to the two proline-rich motifs site in the PDCoV N protein were instructed, including mPDCoV-N(P61A), mPDCoV-N(P292A) and mPDCoV-N(P61A/P292A). Sequence alignment of PDCoV N proteins was finished by Clustal X. Mutations residues are shown in an arrow. (**b**) The recombinant PDCoV N and Grb2 proteins were expressed and purified for the GST pull-down analysis by Western blotting with an anti-GST and anti-PDCoV N monoclonal antibody. (**c–e**) The binding affinity between mPDCoV N and Grb2 was analysed by QCM.

To identify the essential domains of PDCoV-N that mediate its interaction with Grb2, three mutants of PDCoV N protein based on its two conserved proline-rich motifs were generated. In detail, the proline-rich motifs PIPP at the N terminus of PDCoV N spanning 61 to 64 aa and the proline-rich motifs PPTKP at the C terminus of PDCoV N spanning 291 to 295 aa were mutated alone or simultaneously into AIPP and PATKP through changing proline (P) to alanine (A). The three mutants were respectively named mPDCoV-N (P61A), mPDCoV-N (P292A) and mPDCoV-N (P61A/P292A) (Fig. 6a). The ability of three mutants to bind to Grb2 was assessed using GST pull-down assay (Fig. 6b). The results showed that the knockout of one out of two proline-rich motifs decreased the binding affinity, compared with that of the WT wPDCoV-N. However, the binding affinity of the knockout of two proline-rich motifs protein named mPDCoV-N (P61A/P292A) was higher than the knockout of one out of two proline-rich motifs but less than WT. It demonstrates that the proline-rich motifs in N- or C-terminal region of PDCoV N both were critical for the interaction between PDCoV-N and Grb2.

In addition, the QCM test was carried out to confirm the essential domains to compare the binding affinity between Grb2 and WT or mutated PDCoV-N. The results showed that the interaction between wPDCoV-N and Grb2-GST was stronger than that between three mPDCoV-N and Grb2-GST, and the binding ratio from high to low was wPDCoV-N (31.58 %), mPDCoV-N (P61A/P292A) (19.67 %) (Fig. 6e), mPDCoV-N (P61A) (6.38 %) (Fig. 6c) and mPDCoV-N (P292A) (1.75 %) (Fig. 6d). These results indicated that the knockout of the two proline-rich motifs of PDCoV-N could not completely disrupted the interaction between PDCoV-N and Grb2, but can drastically decreased interaction between them. However, we cannot rule out the possibility that interaction between mutated PDCoV-N and Grb2 has other ways by the structure of N protein or other motifs.

### PDCoV-N promotes Grb2 degradation by ubiquitin-proteasome pathway

**Fig. 7.**
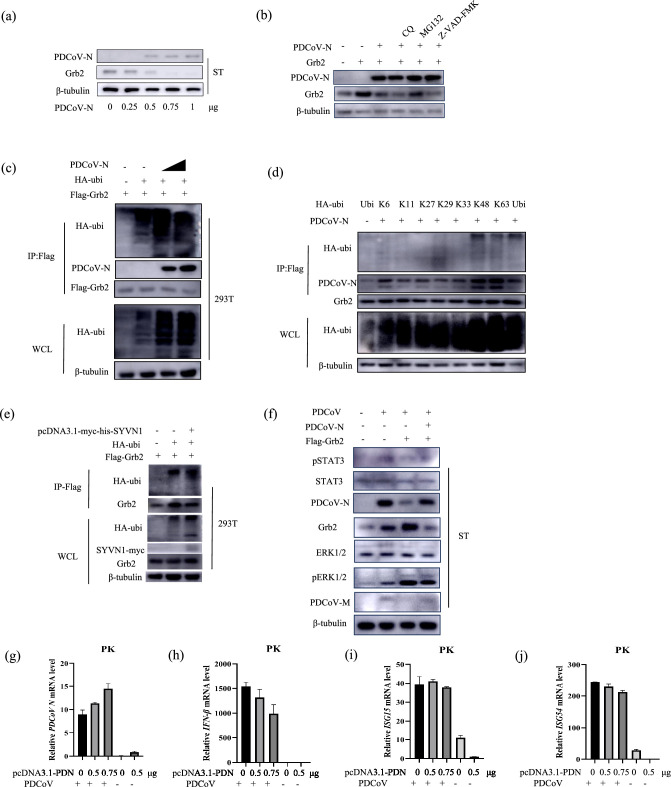
PDCoV-N promotes Grb2 degradation by ubiquitin-proteasome pathway (**a**) ST were transfected pcDNA3.1-PDN plasmid with increasing concentrations (0, 0.25, 0.5, 0.75 and 1 µg). Western blotting was used to analyse expression. (**b**) ST cells were transfected with plasmid encoding Flag-pGrb2, PDCoV N or the empty vector for 18 h and then were pretreated with DMSO, MG132 (10 mM), V-ZAD-FMK (10 mM) or CQ (50 mM) for 2 h. PDCoV N and Grb2 expression were measured after 12 h. (**c**) 293 T cells were cotransfected with Flag-tagged Grb2, different dose PDCoV N, and HA-tagged ubiquitin (HA-Ubi) or empty control plasmids. The cells were lysed for Co-IP by Flag-affinity gel after 24 h. (**d**) 293 T cells were co-transfected with Flag-tagged Grb2 and PDCoV N or an empty control plasmid with HA-Ubi, K6, K11, K27, K29, K48 and K63. The cells were lysed for Co-IP by Flag-affinity gel. (**e**) 293 T cells were co-transfected with Flag-tagged Grb2, HA-ubi and Myc-tagged SYVN1 for 24 h. The cells were lysed for Co-IP by Flag-affinity gel. (**f**) The ST cell was transfected with plasmids pcDNA3.1-pGrb2, pcDNA3.1-PDN or pcDNA3.1 for 24 h and infected PDCoV at 0.01 MOI. The expression of candidate proteins was analysed. (**g–j**) RT-qPCR was used to detect the expression of IFN-β, ISG15 and ISG54 on different doses of pcDNA3.1-PDN and then were infected PDCoV for 12 h.

PDCoV-N protein is mass-produced in infected cells and plays an important role in enhancing the efficiency of viral replication. PDCoV N is capable of interacting with Grb2. So, we investigated the effect of PDCoV N on Grb2 expression. ST cells were transfected with plasmids pcDNA3.1-PDN with different doses. The results showed the N protein strongly reduced the endogenous Grb2 expression in ST cells in a dose-dependent manner (Fig. 7a). At present, it has been reported that there exit three mainly protein degradation pathways, including ubiquitin-proteasome, autolysosome and apoptosis pathway. To further identify the predominantly mediates way, ST were transfected with plasmids pcDNA3.1-PDN and then were treated with the autophagy inhibitor chloroquine (CQ), the protease inhibitor MG132 or the caspase-family inhibitor Z-VAD-FMK. Ubiquitin-proteasome inhibitor MG132 treatment reversed PDCoV N degrades-mediated Grb2 degradation, autophagy inhibitor CQ and caspase-family inhibitor Z-VAD-FMK did not, indicating PDCoV N protein mediated the degradation of Grb2 through the ubiquitin-proteasome pathway (Fig. 7b).

HEK293T cell was cotransfected with Flag-tagged Grb2, PDCoV N or empty vector expression plasmids, together with the HA-tagged ubiquitination plasmids for 24 h. The results revealed that PDCoV N protein increased the polyubiquitination of Grb2 in dose-dependent (Fig. 7c). The plasmids expressing different ubiquitin mutants retaining only a single lysine residue (K6, K11, K27, K29, K33, K48 and K63) were used to determine the type of ubiquitination modification. Western blotting results indicated that K48- and K63-linked polyubiquitination of Grb2 remarkably increased after PDCoV N protein expression (Fig. 7d). Co-IP assay with LC-MC/MS was performed using Flag and PDCoV N as the bait (Table S1). E3 ubiquitin ligase SYVN1 interacts with both PDCoV N and Grb2 proteins. We speculated that PDCoV-N degraded Grb2 under the action of SYVN1. To investigate whether SYVN1 participates in the ubiquitin process, ST cells were transfected with pcDNA3.1-SYVN1-myc-his or pcDNA3.1-myc-his for 24 h. Then, Co-IP assay was performed using anti-Flag agarose beads and detected with anti-Flag, anti-PDCoV N and anti-myc MAb. As shown in Fig. 7e, SYVN1 promoted the ubiquitin of Grb2 degradation. However, SYVN1 were not coimmunoprecipitation with Flag-Grb2 and PDCoV N, perhaps due to the weaker sensitivity of Western blotting compared with mass spectrometry.

To investigate whether PDCoV N participates in the regulation of Grb2 activation, the Grb2-overexpressing ST cells were cotransfected with plasmids pcDNA3.1-PDN or empty vector plasmid and then infected with PDCoV. The replication effect was assessed at 18 hpi by Western blotting (Fig. 7f). The results revealed that the viral protein PDCoV N and M were significantly increased in PDCoV-N-overexpressing ST cells compared with their levels in empty vector. The level of pERK1/2 dramatically decreased, while the pSTAT3 protein increased in PDCoV-N overexpressed ST cells. Next, IFN-β productions were measured by PDCoV-N induced. On the basis of the rise of plasmid (pcDNA3.1-PDN) quantity, the IFN-β, ISG15 and ISG54 mRNA levels notably lowered (Fig. 7g). PDCoV N inhibits the IFN-β (Fig. 7h), ISG15 (Fig. 7i) and ISG54 (Fig. 7j) production. These results reveal that PDCoV N inhibits the activation of Grb2.

### The other coronavirus N protein does not interact with Grb2

**Fig. 8.**
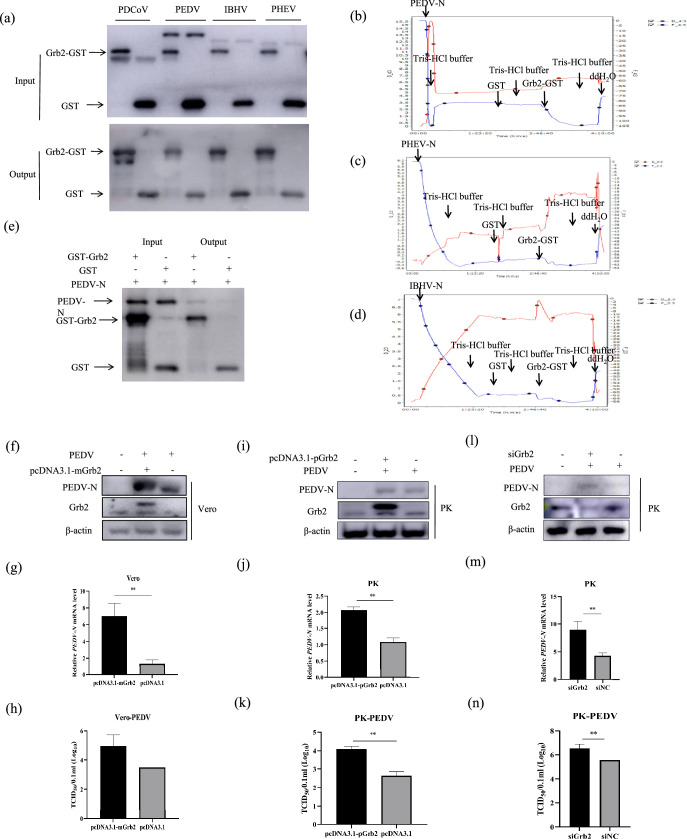
Grb2 increased PEDV replication in LLC-PK1 and Vero cells. (**a**) The full length of the PDCoV N gene with 6×Hig-tagged and its homologs include *Alphacoronavirus* genus PEDV (MF152604), *Betacoronavirus* genus PHEV (ARC95278.1) and *Gammacoronavirus genus* IBHV (NP_040838.1) were expressed and purified for GST pull-down analysis. (**b–d**) The binding affinity between other coronavirus N proteins and Grb2 were measured by QCM. (**e**) The interaction between PEDV N and Grb2 proteins was measured by GST pull-down. (**f–h**) The monkey-derived Grb2 overexpression plasmid named pcDNA3.1-mGrb2 was transfected into Vero cells and then infected with PEDV for 24 h. Whole-cell lysates were assayed by Western blotting and RT-qPCR. LLC-PK1 cells were transfected with pcDNA3.1-pGrb2 (**i–k**) or siGrb2 (**l–n**) and the empty vector and infected with 0.1 MOI PEDV and harvested at 36 hpi. PEDV N expression was analysed with Western blotting, RT-qPCR and TCID_50_.

Generally, the structural protein N is most conserved for all coronavirus. The interaction between Grb2 and the other three subgenera (*Alphacoronavirus*, *Betacoronavirus* and *Gammacoronavirus*) was investigated using GST pull-down and QCM assays. As shown in Fig. 8a, only the N protein deriving from *Deltacoronavirus* PDCoV could directly interact with Grb2. Further, the QCM test showed that the interaction between PDCoV N and Grb2 was stronger than the other three coronavirus (PEDV, AIBV and PHEV). The binding ratio of PEDV-N (Fig. 8b), PHEV-N (Fig. 8c), IBHV-N (Fig. 8d) and PDCoV-N with Grb2 was 23.51%, 8.20%, 5.08% and 31.58%, respectively. It is worth noting that PEDV-N showed a limited binding affinity with Grb2 in the QCM test. GST pull-down was finished to further investigate the relationship (Fig. 8e). The N protein deriving from *Alphacoronavirus* PEDV could also interact with Grb2. This result indicats that PEDV-N may interact with Grb2 slightly.

Grb2 can regulate *Deltacoronavirus* PDCoV replication. To further investigate whether Grb2 participates in the regulation of PEDV replication, the plasmids pcDNA3.1-pGrb2 (Fig. 8i–k) and siGrb2 (Fig. 8l–n) were transfected into LLC-PK1, and then the cells were infected with PEDV. Vero was also chosen to study by transfecting with pcDNA3.1-mGrb2 (Fig. 8f–h). The results revealed that the PDCoV N protein and mRNA levels were both significantly increased by Grb2-overexpressing LLC-PK1 and Vero cells compared with their levels in empty vector plasmid transfected cells. RT-qPCR and Western blotting results showed that Grb2 regulated PEDV replication in LLC-PK1 and Vero cells at indicated time points. The overexpression of Grb2 increased PEDV replication in LLC-PK1 and Vero cells. Downexpression of Grb2 also significantly increased PEDV N mRNA and protein abundance in LLC-PK1 cells during PEDV infection. The TCID_50_ showed the upregulation was statistically significant. The knockdown of Grb2 also increased the PEDV replication in LLC-PK1 cells. To summarize, the PEDV N protein of *Alphacoronavirus* could interact with Grb2, while the N protein of *Betacoronavirus* AIBV and *Gammacoronavirus* PHEV could not. Grb2 upregulated PEDV replication.

## Discussion

MAPK is the major downstream pathway by adaptor protein Grb2 [37]. Viruses have evolved several methods for optimizing infection with the help of MAPK signalling [32,38]. In our study, the ST cell with PDCoV-infected upregulated Grb2 and induced activation of ERK1/2/MAPK. The ERK activity plays a pivotal role in viral biosynthesis. However, the overexpression of Grb2 inhibited PDCoV replication in ST cells. We speculate that ERK1/2 was excessive activation as continuous upregulation of Grb2, which increased IFN-β production and induced inhibition of PDCoV replication. To suppress production of IFN-β, the continued increasing PDCoV N reduced the endogenous Grb2 in a dose-dependent manner to downregulate the excessive activity of ERK1/2. That’s why the expression of Grb2 in LLC-PK1 cells infected with PDCoV shows a trend from increasing to decreasing. Early research also confirmed my hypothesis. Jeon observed the comparably dynamic activation state of ERK1/2 in cells infected with PDCoV. The activity of ERK1/2 was increased at 3 hpi, with a peak at 6 hpi and a dramatic decrease at 9 hpi. The gradually increased level of Grb2 in ST cells infected with PDCOV, due to the replication rate of PDCoV in ST cells, is much lower than in LLC-PK1. Low expression levels of N protein cannot inhibit the expression of Grb2. Xu found that ERK activation negatively regulated B19V infection [29]. Freed also found that MEK/ERK is a negative regulator of antiviral gene expression by limiting expression of IFN-β [39]. MEK/ERK may have opposing roles in the context of different viruses or different target cells. As viruses often encode for proteins to interfere with host innate immune signalling, these results are sometimes difficult to generalize [39]. In this study, we first demonstrated that PDCoV infection increased Grb2 expression, which may suggest the regulatory functions of Grb2 under virus stimulation. Then, we found that overexpressed Grb2 induced activation of ERK1/2/MAPK in PDCoV-infected cells and inhibited viral production.

JAK-STAT are the most prominent innate immune signalling pathways. Battcock studied that the Ras/Raf/MEK pathway downregulates IFN-induced antiviral response. The activation of the Ras/Raf/MEK pathway may be one defining factor of cellular sensitivity to IFN action [40]. Stat1 and Stat2 are often used as the focus of IFN research. The other STATs include Stat3, which regulates important processes of some viruses but poorly defined [41]. STAT1 and STAT2 heterodimers, supporting a potential antiviral role, have been described to bind regulatory elements promoters of the expression of ISG and GAS [42]. STAT1 and STAT3 heterodimers as negative feedback have been proposed that can effectively quench STAT1 in a later phase of the IFN response [42]. Receptor tyrosine kinases could activate the Stat3 signalling pathway via a Grb2-/Gab2-dependent mechanism in Friend erythroleukemia virus infected [43]. Zhang demonstrated that Grb2 downregulates Stat3 activation as Grb2 inhibits the interaction between Stat3 and EGFR by competitive binding to the EGFR [44]. Viruses escape natural immunity by regulating STAT3 activity. PRRSV nsp5 inhibits the OSM-activated gp130/JAK/STAT3 signalling by proteasome degradation for virus replication and spread [45]. In our studies, Grb2 increased PDCoV -induced IFN-β activation by MAPK-ERK1/2-STAT3.

To combat the antiviral effects of IFN, both structural and nonstructural proteins of PDCoV have evolved various mechanisms. PDCoV N protein interacted with RIG-I’s CTD and helicase domain to regulate IFN-β production [46]. Ji demonstrated that PDCoV N protein promoted porcine IRF7 degradation via the ubiquitin-proteasome pathway to increase the K6-, K11- and K29-linked polyubiquitination of porcine IRF7 [47]. The N-terminal region (1-246) of PDCoV N protein was associated with pRIG-I K63-linked polyubiquitination to impede from binding double-stranded RNA directly [20]. In our studies, PDCoV N degrades Grb2 under the action of SYVN1 by proteasome pathway to promote virus replication. As an inhibitor of IFN-β production, the CoV N protein exists in various mechanisms in different CoVs and hosts [47].

The study not only first demonstrated the regulator mechanism of Grb2, but clarified the relationship sites between PDCoV nucleoprotein and host Grb2. Grb2 can interact with the proline-rich (PR) domain including P**P*R for nSH3 (region 61–64 aa) and P***P**KP for cSH3 (region 291–295 aa) (* represents any natural amino acid) [48]. In our study, the two conserved proline-rich motifs of PDCoV-N are important for binding to Grb2, but two proline-rich motifs of PDCoV-N could not completely disrupt the interaction. The binding ratio of mutant containing 61 and 292 two sites was much higher than only sites 61 and 292 but less than the wild-type. Similarly, one study found that nSH3/cSH3 exhibit mutually exclusive binding to the SOS1 peptide PVPPPVPPRRRP, but they are all strong binding if alone [48]. The results showed the importance of tertiary structure in determining protein function. The rigidity of the proline-enriched region may be high, and there are many irregular regions; in the other regions of PDCoV-N, they have a relatively high impact on the structure after mutation. The special function of PDCoV N protein may be its particular structure. The mutated PDCoV-N binds to Grb2 differently. *In vivo*, PDCoV N interacts with Grb2 through proline-rich motifs to disrupt the ERK1/2 signal, which mediates the upregulation of PDCoV replication. Early studies have shown that the 11KD protein of the B19V virus inhibits ERK activation and regulates virus replication by disrupting the binding of SOS with Grb2, a new mechanism of PDCoV immune escape through nucleoprotein protein. A comprehensive and systematic understanding of the host cellular responses to different CoVs infections will provide insights for novel drug development efforts against CoVs disease. Here, we chose four strains of CoVs, including PEDV, PHEV, AIBV and PDCoV representing *Alphacoronavirus*, *Betacoronavirus*, *Gammacoronavirus* and *Deltacoronavirus* genera, respectively. This study demonstrates a contrasting, similar and unique mechanism exploited by CoVs interacting with Grb2. Altogether, different viruses also develop immune escape mechanisms according to their circumstances.

**Fig. 9.**
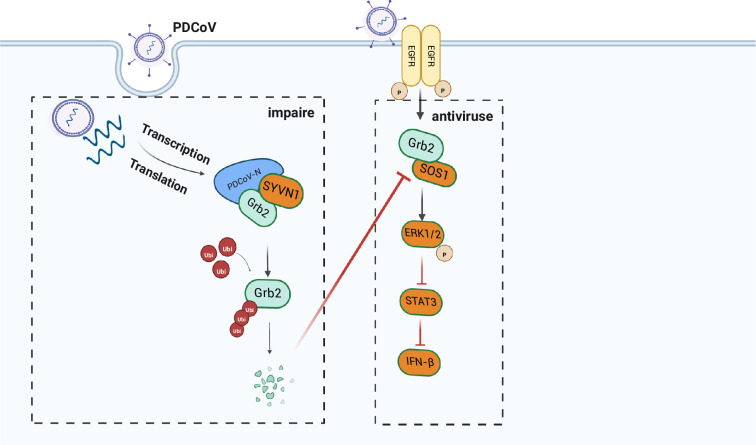
The antiviral mechanism of Grb2 inhibits PDCoV replication. During PDCoV infection, Grb2 promotes IFN-β production through activating Raf/MEK/ERK/STAT3 signalling pathway in PDCoV-infected ST cells to suppress viral replication. PDCoV N is capable of interacting with Grb2 by the proline-rich motifs in the N- or C-terminal region of PDCoV N. PDCoV N promotes Grb2 degradation by K48- and K63-linked ubiquitin-proteasome pathways to inhibit the effect of Grb2.

In summary, we have shown that overexpression of Grb2 downregulates the expression of STAT3 and activates IFN-β through the ERK1/2/MAPK pathway to decrease viral replication. PDCoV N is capable of interacting with Grb2 by the proline-rich motifs in the N- or C-terminal region of PDCoV N. Besides that, PDCoV N promotes Grb2 degradation by K48 and K63-linked ubiquitin-proteasome pathways to escape immunity (Fig. 9) (created with BioRender.com). Our study demonstrates a new function of Grb2-mediated viral restriction by ERK1/2-STAT3-IFN-β pathway cascades and insight into how PDCoV degrades Grb2 protein to promote replication. The study offers new clues for treatment and vaccine development against PDCoV.

## Supplementary material

10.1099/jgv.0.002014 Uncited Table S1.

## Abbreviations

CoVs: coronaviruses
CQ: chloroquine
ERK1/2: extracellular signal-regulated kinases 1 and 2
GEF: guanine nucleotide exchange factor
Grb2: growth factor receptor-bound protein 2
GST: glutathione S-transferase
hpi: hour post-infection
LLC-PK1: porcine kidney cells
MAPK: mitogen-active protein kinase
MOI: multiple of infection
nsps: nonstructural proteins
ORF: open reading frame
PDCoV: porcine deltacoronavirus
pERK1/2: phosphorylation of ERK1/2
QCM: quartz crystal microbalance test
RTKs: receptor tyrosine kinases
RT-qPCR: real-time quantitative PCR
SH2/3: Src homology 2/3
siNC: non-targeting siRNA
siRNA: short interfering RNA
ST: swine testis cells
TCID: tissue culture infective dose
TMT: tandem mass tag
Vero: African green monkey kidney cells
